# Real Estate Prices, Inflation, and Health Outcomes: Evidence From Developed Economies

**DOI:** 10.3389/fpubh.2022.851388

**Published:** 2022-02-14

**Authors:** Wensheng Bao, Ran Tao, Anees Afzal, Hazar Dördüncü

**Affiliations:** ^1^Department of Jiaozhou Campus Construction, Qingdao University, Qingdao, China; ^2^Qingdao Municipal Center for Disease Control and Prevention, Qingdao, China; ^3^Department of Accountancy, Superior University, Lahore, Pakistan; ^4^Department of International Trade and Logistics, Faculty of Economics, Administrative and Social Sciences, Nisantasi University, Istanbul, Turkey

**Keywords:** real estate, prices, inflation, health, life expectancy, GDP

## Abstract

Population health is a key pillar of the fast-growing economies, but several challenges threaten it. This study scrutinizes the impact of real estate prices (housing rent) and inflation on population health by using advanced economies from 1996 to 2019. Health is measured by infant mortality rates and life expectancy at birth. The empirical outcomes show a positive and significant effect of housing rent on the infant mortality rate. In contrast, housing rent improves life expectancy. We also find that an increase in inflation positively affects the infant mortality rate and has a negative effect on life expectancy. GDP and health expenditure tend to improve health by increasing life expectancy and reducing the infant mortality rate. However, unemployment is harmful effects on population health. This study recommends that healthcare practitioners consider the housing market and inflationary pressure.

## Introduction

Since the last 20 years, many health economists have immensely debated the causal association between health and wealth. It is found that there exists a positive association between health and wealth, but the causality direction is still not clear (1). The studies were done by Apouey and Clark (2), Arellano and Bond (3), and Atalay et al. (4) consider inheritances and lottery wins as sources of variation in wealth to examine the linkage between health and wealth. Another strand of literature considers house prices as an exogenous source to explore the impact of wealth on health. Ratcliffe et al. (5) reveal that an upsurge in house prices positively influences mental health in the UK. The study also claims that this effect could also be due to available economic opportunities and amenities. Fichera and Gathergood (6) reported that an increase in house price positively impacts non-chronic and general health conditions of house owners in the UK. Recently, studies have also reported impacts of mortgage indebtedness and foreclosure on the health conditions of households (7–9).

Although there is vast unanimity regarding the association between household wealth, house prices, and households' health, there is limited consensus on its causes. Literature categorizes three major channels through which an upsurge in house prices and wealth influences the health of individuals. The first channel denotes that an unanticipated upsurge in wealth tends to increase consumption level, reduce working hours, and upsurge healthcare investment of house owners (10, 11). It is expected that the wealth effect is larger for outright owners as compared to mortgage owners. In the case of mortgage owners, the wealth effect is offset by mortgage repayments. The mortgage owner has to trade-off in many ways based on repayment magnitudes, such as reducing consumption or leisure, increasing working hours, or compromising housing suitability and quality. Literature reveals that large mortgage debts homeowners have to work longer hours (4, 12, 13). It advocates that mortgages constraint may not permit translation of the effects of wealth on health investment. In contrast, renters seek shock of house price increase in two ways. Firstly, rentals receive increased payments of rent if a high correlation exists between rental rate and house prices. Secondly, if renters want to attain ownership, the high prices of houses will create hurdles in such transitions. Literature shows that housing stress and affordability have a detrimental impact on the mental health and financial wellbeing of house owners (14, 15).

The second channel is that economic prospects and local amenities are major determinants of the relationship between health, house prices, and wellbeing. House prices determine the value of amenities such as the extent of safety, proximity to services/transport/schools, and community involvement. Thus, house prices are positively associated with individuals living in this area, and the individuals attaining better amenities are healthier both psychologically and physically. Meanwhile, prosperous areas enrich with better conditions of the labor market and business growth command high property prices. Moreover, areas with good macroeconomic conditions ensure lower uncertainty of income and higher expectations of income. The individuals of local communities experience the same economic opportunities and quality of facilities, thus, experiencing the same level of health outcomes. It is suggested to control the local area's economic conditions and amenities to deduce the true impact of wealth and changes in house rents on health outcomes.

The third channel states that reverse causality occurs between health and house rent if poor individuals are forced to relocate to lower house rental areas. In contrast, households with healthy psychological and physical conditions attain high income and are optimistic regarding the selling price of their properties and employment opportunities in the future. Resultantly, they prefer to settle in those areas where more employment opportunities are available, and house prices are relatively higher. Thus, the moving activity creates a high correlation between house rents and health.

Another major aspect that influences the health outcome is persistently increasing inflation. In the wake of rising and high inflation, children and females are at risk of poor health and malnutrition in developing economies. In view of the United Nations, the mortality rate for children under the age of five is recorded at almost 5.4 million during 2017 (United Nations, 2018). Several studies reported the influence of inflation on child mortality. However, the implications of inflation on health outcomes are yet unexplored (16). Due to constraints in budget and rising inflation, households have to compromise on nutritional quality which results in malnutrition, child mortality, and poor health conditions. Empirical studies report that inflation shocks might affect education, social behavior, outcomes of the labor market, health, and skills formation (17, 18). During inflation, the resulting decline in affordability influences the wellbeing of people through several channels, such as a reduction in investment for education, lower levels of consumption, malnutrition, and poor health outcomes.

The nexus between real estate prices and health is still puzzling, but the transmission channels are not clear among inflationary pressure and human health. We use the hedonic pricing theory framework to integrate health and urban sociological schools of thought. Factors that affect human health, such as healthy food access, alcohol and tobacco, crime, real estate industry, macroeconomic issues, and social capital (19). Previous studies usually ignored the link between real estate prices and health outcomes in developed economies. The researches on the effects of inflationary pressure on human health in developed countries are still limited and important. Some research works provide country-specific empirical evidence which cannot be generalized for the advanced nations. This study is novel of its kind that offers an empirical analysis of real estate prices, inflationary pressure, and health using advanced economies panel data set. The possible issue of endogeneity is also fixed using instrumental econometric approaches. This study offers novel findings on the real estate industry and health is based on qualitative studies.

From the above discussion, it is obvious that house rents and inflation directly influence the population's health outcomes. An accurate investigation is required to design programs and policies for the wellbeing of people. To the best of the authors' knowledge, this pioneering study attempts to explore the combined effects of house rents and inflation on health outcomes. The study intends to investigate the impacts of house rents and inflation on the health outcomes of developed economies by using panel data approaches. This study reformulates fragmented research on this research theme and provides robust implications. This study offers some new policy implications which could help in improving health in developed economies.

## Model, Methods, and Data

A bulk of the literature in health economics includes empirical studies that have tried to assess the impacts of house prices and inflation on health outcomes. Therefore, following the standard literature (4, 20), we adopt the following econometric model:


$$\begin{array}{l} Health_{it}=\;\varphi_{0}+\varphi_{1}HR_{it}+\varphi_{2}Inflation_{it}+\varphi_{3}GDP_{it}+\varphi_{4}HE_{it}\\ \;+\varphi_{5}Unemp_{it}+\alpha_{i}+\varepsilon_{it} \end{array}$$

Where *Health*_*it*_ is the dependent variable for nation i at period t, φ_0_ is the constant term, α_*i*_ is an unobserved specific effect, and ε_*it*_ is an error term. Where *Health*_*it*_ is the health production measured by house rent (HR), inflation rate (inflation), GDP per capita (GDP), health expenditure (HE), and unemployment (Unemp)? If housing price is to boost the health of owners, an estimate of φ_1_ is expected to be positive, and if housing rent negative impact on the health of renters, an estimate of φ_1_ is to be negative. We also noted that inflation has a significant harmful effect on health, an estimate of φ_2_ is to be negative. GDP per capita and health expenditure also have a favorable impact on health outcomes. Thus estimates of φ_3_ & φ_4_ should be positive. An increase in unemployment is also expected to raise social and economic problems and reduce health. The model outlined by specification (1) is a panel data model, and it is estimated by fixed effects (FE) and random effects (RE) methods. The dilemma of FE or RE models can be solved via the Hausman test, one of the panel model's classical tests.

Due to the endogeneity problem, the baseline specification is also estimated with two-stage least squares (2SLS). The first three estimation methods are static panel techniques, but the generalized method of moments (GMM) is a dynamic panel data approach that allows us to include the lagged level of health outcomes. Previous literature reported that the level of current health status is strongly affected by the level of health status in the previous year, so our augmented model is written as:


$$\begin{array}{l} Health_{it}=\;\varphi_{0}+\lambda_{1}Health_{it-1}+\;\varphi_{1}HR_{it}+\varphi_{2}Inflation_{it}\\ \;+\varphi_{3}GDP_{it}+\varphi_{4}HE_{it}+\varphi_{5}Unemp_{it}+\alpha_{i}+\varepsilon_{it} \end{array}$$

GMM uses a set of instrumental variables to solve the problem of endogeneity. The GMM estimators can be estimate coefficients via difference and system (3, 21). At least, there are two main reasons for choosing GMM. The first is to control for country-specific effects, and the second is to control for endogenous problems. Another edge of the GMM method is that it also captures time-series variation in the data and allows for the addition of lagged dependent variables as repressors. Using panel standard methods of estimations, we assess the impacts of house price and inflation on health outcomes in the next section. Previous literature has used the same methods for health outcomes (22).

The study aims to explore the impact of house rents and inflation on the health outcomes of developed economies (Canada, France, Japan, Netherlands, Spain, Switzerland, Sweden, United Kingdom, USA) from 1996 to 2019. Descriptive statistics and details regarding symbols, definitions, and sources of data are given in Table 1. Health outcome is measured by infant mortality rate (per 1,000 live births) and life expectancy at birth in total years. The focused explanatory variables are house rents and inflation. House rent prices are taken at the base of 2015. At the same time, consumer prices in annual percentage are used to measure inflation. Besides focused variables, GDP per capita (at 2015 US$), health expenditures as a percent of GDP, and unemployment (in percent of the total labor force) are used as control variables. Data for house rent is extracted from the OECD, while data is taken from the World Bank for the remaining variables. Table 2 shows the correlation matrix of variables. It is obvious from the correlation matrix that all explanatory variables are free from multicollinearity problems.

**Table 1:** Descriptive statistics.

**Variables**	**Mean**	**Std. Dev**.	**Min**	**Max**	**Definitions**	**Sources**
IM	4.176	1.265	1.800	7.700	Mortality rate, infant (per 1,000 live births)	World Bank
LE	80.49	1.902	76.02	84.35	Life expectancy at birth, total (years)	World Bank
HR	88.28	13.27	57.26	115.7	House rent prices, 2015 = 100	OECD
Inflation	1.479	1.145	−1.334	4.156	Inflation, consumer prices (annual %)	World Bank
GDP	10.63	0.314	9.925	11.39	GDP per capita (constant 2015 US$)	World Bank
HE	9.957	2.343	5.853	17.47	Current health expenditure (% of GDP)	World Bank
Unemp	7.207	4.262	2.120	26.09	Unemployment, total (% of total labor force) (modeled ILO estimate)	World Bank

**Table 2:** Correlation matrix of variables.

	**IM**	**LE**	**HR**	**Inflation**	**GDP**	**HE**	**Unemp**
IM	1						
LE	−0.844	1					
HR	−0.573	0.797	1				
Inflation	0.418	−0.447	−0.393	1			
GDP	0.181	0.059	0.153	−0.175	1		
HE	0.322	−0.021	0.314	0.022	0.511	1	
Unemp	−0.137	0.088	−0.044	0.074	−0.598	−0.188	1

## Results and Discussion

The study adopts the fixed effect model (FEM) and random effect model (REM) methods to extract empirical findings of health outcomes. Table 3 provides the results of the FEM and REM models. To capture the issue of endogeneity, the study uses GMM and 2SLS estimation approaches. Table 4 reports the empirical results of GMM and 2SLS models. There are four models in Tables 3, 4. In Table 3, the infant mortality rate is the dependent variable in model 1 and model 2, while life expectancy is the dependent variable in model 3 and model 4. In Table 4, the infant mortality rate is the dependent variable in model 1 and model 3, while life expectancy is the dependent variable in model 2 and model 4.

**Table 3:** House rent, inflation, and health (FEM and REM).

	**Infant mortality rate**		**Life expectancy**	
	**FEM**	**REM**	**FEM**	**REM**
HR	−0.020^**^	−0.027^***^	0.040*	0.089^***^
	(0.009)	(0.008)	(0.022)	(0.017)
Inflation	0.002	0.001	−0.026	−0.011
	(0.020)	(0.015)	(0.040)	(0.037)
GDP	−1.028	−0.170	6.815^**^	3.076^**^
	(1.107)	(0.708)	(2.690)	(1.656)
HE	−0.214^**^	−0.194^*^	0.328^*^	0.303^*^
	(0.082)	(0.117)	(0.181)	(0.161)
Unemp	0.011	0.004	−0.111^**^	−0.099^*^
	(0.012)	(0.013)	(0.042)	(0.051)
Constant	19.10	10.38	1.155	59.66^***^
	(11.12)	(6.948)	(26.73)	(16.47)
Hausman-test	3.69^*^		7.65^**^	
Observations	216	216	216	216
R-squared	0.892		0.877	
Number of code	9	9	9	9

*Robust standard errors in parentheses*.

*** *p < 0.01*,

** *p < 0.05*,

* *p < 0.1*.

**Table 4:** House rent, inflation, and health (2SLS and GMM).

	**Infant**	**Life**	**Infant**	**Life**
	**mortality rate**	**expectancy**	**mortality rate**	**expectancy**
	**2SLS**	**2SLS**	**GMM**	**GMM**
L.Health outcomes			0.960^***^	0.868^***^
			(0.021)	(0.049)
HR	−0.056^***^	0.192^***^	−0.016^**^	0.029^**^
	(0.009)	(0.033)	(0.007)	(0.012)
Inflation	0.058^**^	−0.241^**^	0.028	−0.033^*^
	(0.029)	(0.104)	(0.045)	(0.018)
GDP	−2.529^**^	5.696^**^	−0.455^***^	0.649^**^
	(0.995)	(3.269)	(0.128)	(0.311)
HE	−0.157^***^	0.078	−0.012^*^	0.030
	(0.032)	(0.117)	(0.007)	(0.031)
Unemp	0.025^*^	−0.049	0.009^***^	−0.024^***^
	(0.014)	(0.053)	(0.002)	(0.009)
Year			0.007^***^	0.007^*^
			(0.002)	(0.004)
Constant	−16.25	155.3^***^	−9.934^**^	−10.53
	(9.932)	(35.63)	(4.466)	(24.86)
Observations	216	216	198	198
Number of code	9	9	9	9
Sargan test			0.356	0.523

*Robust standard errors in parentheses*.

*** *p < 0.01*,

** *p < 0.05*,

* *p < 0.1*.

Findings of FEM reveal that house rent brings significant and negative impact on infant mortality rate and a significant and positive impact on life expectancy. It implies that a 1 percent upsurge in house rent reduces the infant mortality rate by 0.020 percent and increases life expectancy by 0.040 percent in the sample of selected economies. However, findings disclose that inflation brings no significant impact on health outcomes in developed economies. GDP reports a significant and positive effect on life expectancy, showing that a 1 percent increase in GDP improves life expectancy by 6.815 percent. It is found that the impact of health expenditure is significant and negative on infant mortality rate and significant and positive on life expectancy. It reports that a 1 percent rise in health expenditures reduces the infant mortality rate by 0.214 percent, while it increases life expectancy by 0.328 percent. Findings show that a 1 percent increase in unemployment reduces life expectancy by 0.111 percent. However, the impact of unemployment is insignificant on the infant mortality rate.

Findings of REM show that house rent is significantly and negatively associated with infant mortality rate and it is significantly and positively associated with life expectancy. These findings show that a 1 percent increase in house rent reduces the infant mortality rate by 0.027 percent, increasing life expectancy by 0.089 percent. A 1 percent rise in GDP brings a significant and positive effect on life expectancy by 3.076 percent. Health expenditures are significant and negative on infant mortality rate, while the impact is significant and positive on life expectancy. Coefficient estimates infer that a 1 percent rise in current health expenditures reduces the infant mortality rate by 0.194 percent and improves life expectancy by 0.3030 percent. Life expectancy reduces by 0.099 percent due to a 1 percent increase in unemployment, as shown by the negative coefficient estimate.

Findings of the 2SLS regression model report that house rent is significantly and negatively associated with the infant mortality rate. However, this association is significant and positive in the case of life expectancy. These findings imply that due to a 1 percent upsurge in house rents, the infant mortality rate reduces by 0.056 percent while life expectancy improves by 0.192 percent. Our findings report that house rent is positively linked with health outcomes as it significantly reduces infant mortality rate and improves life expectancy. These results are supported by the study of Atalay et al. (4), which reports that an upsurge in house prices positively affects the physical health of house owners and negatively influences the mental and physical health of house renters. Another study was done by Fichera and Gathergood (6) for the UK supports our findings, who argued that an increase in house prices is positively associated with non-chronic and general health conditions. It is found that house prices are positively linked with area facilities and health outcomes of residing individuals. People with better facilities experience good quality psychological and physical health. Our finding infers that an increase in house prices directly influences the consumption decision of households regarding drinking, smoking, and food expenditures that changes health outcomes. The impact of changes in health outcomes and house prices are concentrated between low-income tenants and homeowners. A similar result is also reported by Campbell and Cocco (23), who show a significant and positive impact of housing prices on the consumption and health of homeowners (24) found that an increase in house prices significantly reduces anxiety in people and improves their health conditions.

In contrast, inflation brings a significant positive impact on the infant mortality rate and a significant and negative impact on life expectancy. These findings show that a 1 percent rise in inflation increases the infant mortality rate by 0.058 percent and reduces life expectancy by 0.241 percent. Our study reports that an increase in inflation results in an increasing infant mortality rate and a decline in life expectancy. These findings are in line with the studies done by Lee et al. (20), who noted that increased inflation reduces people's purchasing power and adversely affects the structure of consumption leading to malnutrition and poor health condition. This means that inflation could also cause unemployment. Thus, people fail to fulfill the necessities of their children, such as quality food, education, and health needs. The impact of inflation on the health sector could be severe. Inflation results in widening the gap between private and public embellishment and disturbs the quality of life. Regarding control variables, GDP and health expenditures tend to reduce the infant mortality rate while unemployment increases the infant mortality rate. In contrast, GDP improves life expectancy while health expenditures and unemployment produce an insignificant impact on life expectancy.

The findings of GMM models display that house rent reduces infant mortality rate and expands life expectancy. These findings reveal that a 1 percent increase in house rent reduces the infant mortality rate by 0.016 percent and increases life expectancy by 0.029 percent. In contrast, a rise in inflation deteriorates health outcomes, as shown by the coefficient estimate, which depicts that a 1 percent increase in inflation reduces life expectancy by 0.033 percent. However, inflation produces an insignificant impact on the infant mortality rate. GDP and health expenditures reduce the infant mortality rate, but unemployment increases the infant mortality rate. In contrast, GDP improves life expectancy, and unemployment declines life expectancy in GMM models.

## Conclusion and Implications

Health does not only mean the absence of disease or infirmity. It is a situation that represents a complete set of physical, mental, and social wellbeing of a person. A healthy person can perform well his social and economic duties and is considered to be an asset for family, society, and nation. On the other side, a person with a sick body and mind does not efficiently perform his economic and social duties and becomes a liability for society. Wealth or affluence is the most critical contributor to the health status of the people. An increase in wealth allows the people to afford better health facilities, which significantly improves the health status of the people. Another critical factor that can directly impact the health outcome is housing prices. More expensive houses are located in the area with better amenities, including health, education, and transport facilities. Moreover, the rising inflation can increase the cost of health facilities that can negatively impact the health status of the majority of people.

In the light of the above discussion, it is pertinent to estimate the impact of housing prices and inflation on health outcomes. Therefore, this analysis focuses on analyzing GDP, house rent, and Inflation on life expectancy and infant mortality in developed economies. The variables are estimated through FE, RE, 2SLS, and GMM for 1996–2019. The estimates of house rent confirm the negative impact on the infant mortality rate with both FE and RE estimation techniques. Conversely, in the life expectancy model, the estimates of House Rent are positive and significant with FE and RE estimation techniques. However, the estimated coefficients of Inflation are insignificant in infant mortality and the life expectancy model irrespective of FE and RE model. On the other hand, the estimates of GDP are positive and significant in the life expectancy model and insignificant in the model of infant mortality rate.

Further, we have also applied 2SLS and GMM methods to fortify our analysis. The estimates of the 2SLS and GMMS methods confirm the positive impact of house rent on life expectancy and the negative impact of house rent on infant mortality rate. Similarly, the inflation estimate with the 2SLS estimation technique implies the positive effect on life expectancy and negative impact on the infant mortality rate. Nevertheless, the estimated coefficient of inflation is only significant in the life expectancy model with the GMM technique. The estimates of GDP positively impact the life expectancy of developed economies whether we use the 2SLS or GMM technique. Contrariwise, the GDP negatively impacts the infant mortality rate with 2SLS and GMM techniques.

The policy implications based on our findings are as follow. It is widely recognized that huge financial resources are required to improve the health infrastructure of developed economies. The developed economies should focus on real estate development, which would increase the income of a nation and provide necessary funds for the development of health sector infrastructure. On one side, policymakers in developed economies should try to increase the rate of economic growth; on the other side, the policymakers should increase the share of health expenditures in total GDP. Moreover, the government should provide health facilities closer to the living areas, particularly the living areas of poor people, which would uplift the health status of the deprived faction of society. Finally, the government should try to keep inflation under control because it is necessary to provide affordable health facilities and quality food, notably to the vulnerable poor people.

Our research is ignoring mental health, maternal health, physical health, and different diseases as a measure of human health. This study does not explore the impact of real estate prices and inflation on quality of life. Authors should also examine the impact of real estate prices and inflation on the quality of life for developing and developed economies. Also, future research can employ other measures of human health and wellbeing.

## Data Availability Statement

Publicly available datasets were analyzed in this study. This data can be found at: https://data.worldbank.org.

## Author Contributions

WB and RT: conceptualization, software, data curation, and writing—original draft preparation. AA: methodology and writing—reviewing and editing. HD: visualization and investigation. All authors contributed to the article and approved the submitted version.

## Conflict of Interest

The authors declare that the research was conducted in the absence of any commercial or financial relationships that could be construed as a potential conflict of interest.

## Publisher's Note

All claims expressed in this article are solely those of the authors and do not necessarily represent those of their affiliated organizations, or those of the publisher, the editors and the reviewers. Any product that may be evaluated in this article, or claim that may be made by its manufacturer, is not guaranteed or endorsed by the publisher.
